# *BRAF* V600E Mutation of Non-Small Cell Lung Cancer in Korean Patients

**DOI:** 10.3390/medicina59061085

**Published:** 2023-06-04

**Authors:** Hyo Yeong Ahn, Chang Hun Lee, Min Ki Lee, Jung Seop Eom, Yeon Joo Jeong, Yeong Dae Kim, Jeong Su Cho, Jonggeun Lee, So Jeong Lee, Dong Hoon Shin, Ahrong Kim

**Affiliations:** 1 School of Medicine, Pusan National University, Beomeori, Mulgeum-eop, Yangsan 50612, Republic of Korea; doctorahn02@hanmail.net (H.Y.A.); ejspulm@gmail.com (J.S.E.);; 2 Department of Thoracic and Cardiovascular Surgery, Biomedical Research Institute, Pusan National University Hospital, Busan 49241, Republic of Korea; 3 Department of Pathology, Biomedical Research Institute, Pusan National University Hospital, Busan 49241, Republic of Korea; 4 Department of Internal Medicine, Biomedical Research Institute, Pusan National University Hospital, Busan 49241, Republic of Korea; 5 Department of Radiology, Biomedical Research Institute, Yangsan Pusan National University Hospital, Busan 50612, Republic of Korea; 6 Department of Pathology, Seegene Medical Center, Busan 48792, Republic of Korea; gag86@naver.com; 7 Department of Pathology, Biomedical Research Institute, Yangsan Pusan National University Hospital, Busan 50612, Republic of Korea

**Keywords:** lung cancer, B-type Raf kinase (*BRAF*), immunohistochemistry, Ventana VE1 antibody

## Abstract

*Background and Objectives: BRAF* mutational status in resected non-small cell lung cancer (NSCLC) in the Korean population is poorly understood. We explored *BRAF* (particularly *BRAF* V600E) mutational status among Korean patients with NSCLC. *Materials and Methods*: This study included 378 patients with resected primary NSCLC who were enrolled from January 2015 to December 2017. The authors obtained formalin-fixed paraffin-embedded (FFPE) tissue blocks and performed peptide nucleic acid (PNA)-clamping polymerase chain reaction (PCR) for detecting BRAF V600, real-time PCR for detecting *BRAF* V600E, and immunohistochemical analyses using the mutation-specific Ventana VE1 monoclonal antibody. For positive cases in any methods mentioned above, direct Sanger sequencing was additionally performed. *Results:* The PNA-clamping method revealed the *BRAF* V600 mutation in 5 (1.3%) of the 378 patients. Among these five patients, real-time PCR, direct Sanger sequencing detected *BRAF* V600E mutations in three (0.8%) patients. Thus, two cases showed differences in their PNA-clamping and the others. Direct Sanger sequencing of PNA-clamping PCR product was performed for two cases showing negative results on direct Sanger sequencing; both contained *BRAF* mutations other than V600E. All patients harboring *BRAF* mutations had adenocarcinomas, and all patients with V600E mutation exhibited minor micropapillary components. *Conclusions*: Despite the low incidence of the *BRAF* mutation among Korean patients with NSCLC, lung adenocarcinoma patients with micropapillary components should be prioritized in terms of *BRAF* mutation testing. Immunohistochemical staining using Ventana VE1 antibody may serve as a screening examination for *BRAF* V600E.

## 1. Introduction

The *BRAF* gene is responsible for encoding the V-Raf murine sarcoma viral homolog B (BRAF) kinase, which plays a crucial role in cellular signaling, survival and proliferation [1]. *BRAF* gene is located on chromosome arm 7q34 and is composed of 18 exons [2]. BRAF is associated with mitogen-activated protein kinase (MAPK) pathways including the rat sarcoma (RAS), rapidly accelerated fibrosarcoma (RAF), mitogen-activated protein/extracellular signal regulated kinase (MEK), extracellular signal-regulated kinase (ERK), and mitogen-activated protein kinase. Mutations in the *BRAF* gene lead to sustained activation of the MAPK pathway, causing it to become a potential oncogenic driver [1]. Almost 300 different BRAF mutations were discovered in melanoma, colorectal cancer, papillary thyroid carcinoma and non-small cell lung cancers (NSCLCs) [3,4]. In addition, BRAF mutations have been classified into three classes. Class I *BRAF* mutation is RAS-independent and has higher kinase activity even in a monomer state. Class I mutation occurs in the valine residue at amino acid position 600 of exon 15; thus, it includes V600 mutations. Class II *BRAF* mutation has an intermediate kinase activity but should form homodimers to be fully activated. Finally, Class III *BRAF* mutation has an impaired kinase activity that requires RAS activation. Class II and III mutations occur either in the glycine of the G loop in exon 11 or in the activation part in exon 15 [5,6]. According to Owsley et al., Class I *BRAF* mutations represented the majority (62.1%) of all *BRAF* -mutant cases (2.4% of all cancers) in 114,662 different tumor sequencing analyses [7].

Now, dabrafenib (BRAF inhibitor) and trametinib (MEK inhibitor) combination therapy is the preferred first-line therapy for the *BRAF* V600E-mutation-positive lung cancer according to the NCCN (National Comprehensive Cancer Network) guidelines. In the French AcSe program, four patients with V600 non-E mutated lung cancer treated with vemurafenib monotherapy had outcomes comparable to the activity of vemurafenib in the *BRAF* V600E mutation [8]. Consequently, a clinical trial targeting V600 non-E mutation in lung cancer, corresponding to Class I *BRAF* mutation, is ongoing to evaluate the activity of dabrafenib and trametinib (NCT04775095). However, Class II and III *BRAF* mutations are not considered to respond to approved BRAF inhibitors [6,8]. Therefore, the evaluation of *BRAF* V600 of exon 15 mutational status, beyond V600E, could become more important.

According to the NCCN guidelines, real-time polymerase chain reaction (PCR), Sanger sequencing, and next-generation sequencing (NGS) are the most commonly recommended methods for *BRAF* mutation examination and immunohistochemistry, with an anti-BRAF p. V600E-specific monoclonal antibody recommended only after extensive validation.

In this study, *BRAF* V600 mutation, particularly the *BRAF* V600E mutational status, was explored with real-time PCR, peptide nucleic acid (PNA)-mediated clamping PCR, direct Sanger sequencing, and immunohistochemistry, which are relatively more feasible to use than NGS. The clinical and pathologic characteristics of the *BRAF* V600E mutation in non-small cell lung cancers were also investigated. 

## 2. Materials and Methods

### 2.1. Patients, Tissue Specimens, and DNA

This study was performed retrospectively. Three hundred and sixty-eight patients who underwent surgical resection for primary non-small cell lung cancer between 2015 and 2017 at Pusan National University Hospital were included. Among them, five patients had synchronous primary lung cancer. The final cohort was 378 cases of primary non-small cell lung cancers. Formalin-fixed paraffin-embedded (FFPE) tissue blocks, which were made at the time of diagnosis, were used. Clinicopathological data were retrieved from the electric medical records and pathologic reports. Genomic DNA was extracted from FFPE blocks using Maxwell 16 FFPE LEV DNA Purification (Promega corp).

### 2.2. PNA-Mediated Clamping PCR (PNA Clamping PCR)

PNA Clamp *BRAF* mutation detection kit (Seegene, Seoul, Korea) was used. Extracted DNA was mixed with a PNA probe, primers (5′-AAACTCTTCATAATGCTTGCTCTG (forward) and 5′-GGCCAAAAATTTAATCAGTGGA (reverse)). SYBR green PCR master mix and all reactions totaled 20 μL. Real-time PCR reaction was performed according to the manufacturer’s instructions using a CFX96 real-time PCR system (BioRad, Pleasanton, CA, USA). The PNA probe sequences were complementary to wild-type (V600). The PNA probe hybridizes to the wild-type *BRAF* sequence, inhibiting the amplification of the wild-type allele and enhancing preferential amplification of mutant sequences. The positive signal was detected by the intercalation of SYBR green fluorescent dye. The cycle threshold (CT) value was automatically calculated. The delta (ΔCT) value was calculated by subtracting the CT value of a test sample from the standard CT value of a control sample (ΔCT = Standard CT − Sample CT). The cutoff for the presence of mutant was ΔCT of 2. *BRAF* V600 PNA clamping PCR was performed in all 378 cases of non-small cell carcinoma.

### 2.3. Real-Time PCR

The real-time PCR used the Real-Q *BRAF* V600E detection kit (Real-Q; Biosewoom, Seoul, Republic of Korea). Real-time PCR was performed with CFX96 real-time PCR Detection system (Bio-Rad) according to the manufacture’s instruction. The master mixture contained 12.5 μL of the 2X PCR reaction mixture and 2.5 μL of the *BRAF* probe and primer mixture. A total of 15 μL of the master mixture was dispensed into PCR tubes. Then, the extracted DNA of 10 μL (containing 50 ng of DNA) was added to each PCR tube. The sample was considered positive for V600E mutation when both the sample and the internal control were amplified and both CT value of the sample and the internal control were less than 40. If a sample showing the difference between CT value of the sample and the internal control was more than 13, the test was repeated. *BRAF* V600E real-time PCR was performed in all 378 cases of non-small cell carcinoma.

### 2.4. Immunohistochemistry

Immunohistochemistry was performed on the same FFPE block used for molecular testing. An automatic staining device (BenchMark XT, Ventana Medical Systems, Tucson, AZ, USA) was used for staining. All samples were cut into 3 μm thick sections and the sections were deparaffinized in an EZ prep. The slides were pretreated with CC1 (cell conditioner 1, pH8.4 buffer) for 64 min antigen retrieval and followed by pre-primary antibody peroxidase inhibition. Then, the slides were incubated with the Ventana BRAF V600E (VE1) mouse monoclonal primary antibody, and Hematoxylin II ^®^ and Bluing Reagent was used for counterstaining. A sample known to have V600E mutation was used as a positive control. A case was considered to be positive when a signal was present in the cytoplasm [9]. Any nuclear staining was ignored.

### 2.5. Direct Sanger Sequencing

*BRAF* exon 15, which potentially contains the c.1799 T > A transversion mutation, was amplified from genomic DNA by PCR using primers 5′-AAACTCTTCATAATGCTTGCTCTG (forward) and 5′-GGCCAAAAATTTAATCAGTGGA (reverse). Amplification was performed under the following conditions: 1 cycle at 94 °C for 5 min, 40 cycles of denaturation at 94 °C for 30 s, annealing at 63 °C for 30 s, and extension at 72 °C for 30 s; then a final extension at 72°C for 5 min using BioRad C1000 (Pleasanton, CA). After purification of the PCR products, direct bidirectional sequencing was performed using the ABI 3730XL DNA Analyzer. Additionally, direct bidirectional sequencing was repeated using the BRAF PNA clamping PCR product, which is rich in mutant alleles, to detect the variants of low level. Direct Sanger sequencing using extracted DNA from FFPE blocks was performed in 5 cases of any positive results for *BRAF* V600 PNA clamping, *BRAF* V600E real-time PCR and immunohistochemistry for VE1. Particularly, direct Sanger sequencing using PNA clamping PCR product was conducted in cases of discordance in other methods.

## 3. Results

### 3.1. Clinicopathologic Characteristics of Resected Non-Small Cell Lung Cancers

A total cohort of 378 patients with resected non-small cell carcinoma was included in this study. All included patients were Korean. Basic data for included patients are shown in Table 1. Patient age ranged from 36 to 86 years (mean: 66.84 ± 8.76 years). The size of the cancer ranged from 0.9 cm to 10.0 cm (mean: 3.37 ± 1.55 cm). There were 238 males (63.0%) and 140 females (37.0%). The study cohort included 255 cases of adenocarcinoma (67.5%), 91 cases of squamous cell carcinoma (24.1%), 5 cases of adenosquamous cell carcinoma (1.3%), 9 cases of large cell neuroendocrine carcinoma (2.4%), 15 cases of sarcomatoid carcinoma (4%) and others (3 cases, 0.8%). Three hundred and five patients (80.7%) had early-stage disease (stage I and II) and the remaining 73 patients (19.3%) had advanced disease (stage III and IV). Among the patients, 168 (44.4%) patients never smoked, 112 patients (29.6%) were ex-smokers and the other 98 patients (25.9%) were current smokers. Information about an EGFR-activating mutation and ALK fluorescence in situ hybridization (FISH)/ALK D5F3 CDx Ventana immunohistochemistry was retrieved from the prior pathologic reports in electronic medical records. One hundred and twenty patients (31.7%) had EGFR-activating mutations and 11 patients (2.9%) had ALK translocation.

### 3.2. BRAF V600 PNA Clamping and BRAF V600E Real-Time PCR

*BRAF* V600 mutation was detected in five cases (1.3%) using a PNA clamping method among 378 non-small cell carcinoma cases (Table 2 and Table 3). By using BRAF real-time PCR, a BRAF V600E mutation was detected in 3 patients (0.8%) among the total 378 cohort (Table 2 and Table 4), and all these positive cases for real-time PCR had positive results in PNA clamping PCR. There were two discordant cases between PNA clamping and real-time PCR.

### 3.3. Immunohistochemistry for VE1

**Figure 1:**
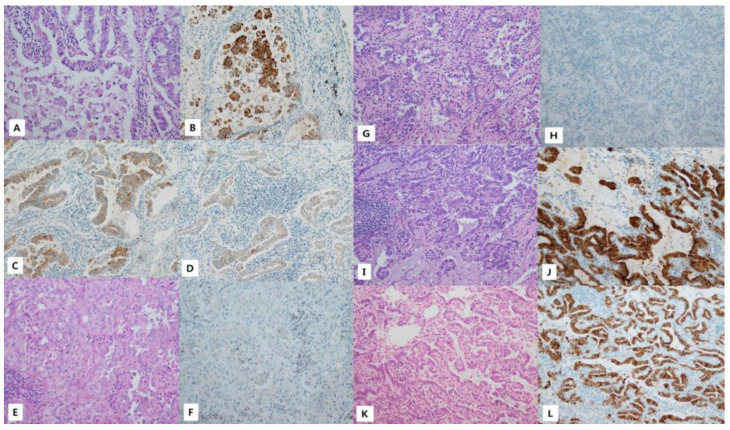
Histologic feature and immunohistochemistry. (**A**–**D**). Patient 142; all ×200. (**A**) A Hematoxylin and eosin (H&E)-stained section showing acinar and micropapillary structures. (**B**) Immunohistochemistry for VE1 (staining intensity 3). (**C**) Immunohistochemistry for VE1 (staining intensity 2). (**D**) Immunohistochemistry for VE1 (staining intensity 1). (**E**,**F**) Patient number 270; all ×200. (**E**) An H&E-stained section showing solid and acinar growth patterns. (**F**) Negative immunostaining for VE1. (**G**,**H**) Patient number 324; all ×200. (**G**) An H&E-stained section showing an acinar growth pattern. (**H**) Negative immunostaining for VE1. (**I**,**J**) Patient number 348; all ×200. (**I**) An H&E-stained section showing an acinar pattern and a few micropapillary structures. (**J**) Immunohistochemistry for VE1 (staining intensity 3). (**K**,**L**) Patient number 358; all ×200. (**K**) An H&E-stained section showing an acinar pattern and a few micropapillary structures. (**L**) Immunohistochemistry for VE1 (staining intensity 2).

Immunohistochemistry for VE1 was performed in all included patients with full-face sections of FFPE blocks. Regarding the results of immunohistochemistry, three patients (0.8%) showed positive staining for tumor cytoplasm (Figure 1, Table 2 and Table 5). All three cases with positive staining showed diffuse positivity for tumor cells. However, two cases had heterogenous staining intensity, though all tumor cells were positive. The other case had diffuse positivity with homogeneous intensity for tumor cytoplasm. The detailed information of staining is shown in Table 5. However, two patients, with positive results for PNA clamping, had negative immunostaining. Regarding the results of immunohistochemistry for VE1, there were two cases showing discordance with the PNA clamping method, and there was no discordant case with real-time PCR. 

### 3.4. Direct Sequencing

**Figure 2:**
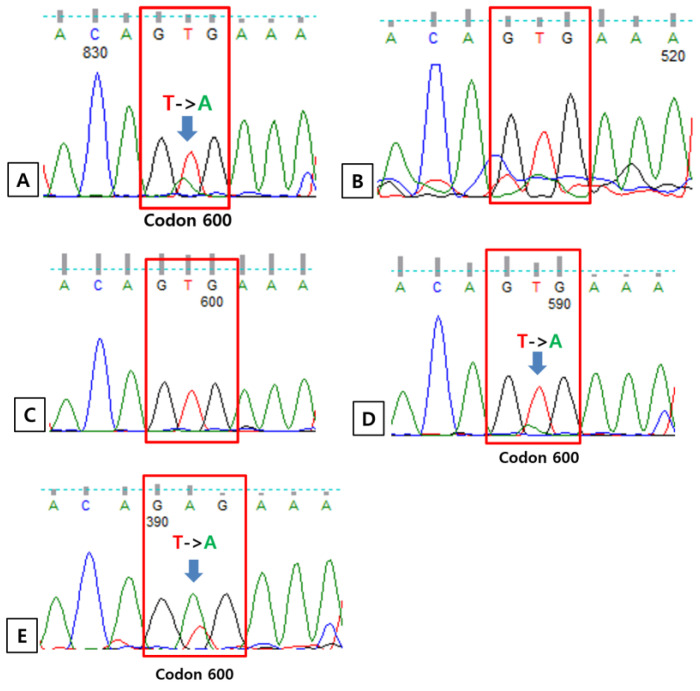
Results of direct Sanger sequencing. (**A**) Case number 142 carries the c.1799 T > A (p. V600E) mutation. (**B**) Case number 270 is wild-type for B-type Raf kinase (BRAF). (**C**) Case number 324 is wild-type for BRAF. (**D**) Case number 348 carries the c.1799 T > A (p. V600E) mutation. (**E**) Case number 358 carries the c.1799 T > A (p. V600E) mutation.

**Figure 3:**
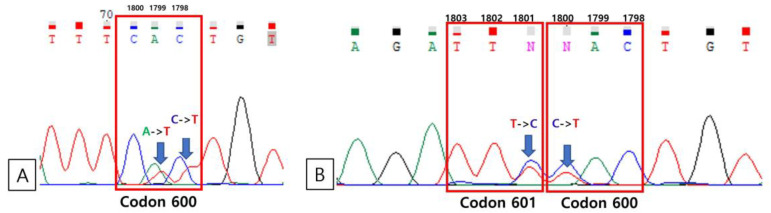
Direct sequencing of peptide nucleic acid (PNA) polymerase chain reaction (PCR) products reveals two p. V600E-negative cases. (**A**) Case number 270 carries the c.1798_1799 CA > TT (p. V600K) for B-type Raf kinase (BRAF) mutation. (**B**) Case number 324 carries the c.1800 C > T (p. V600V) and c.1801 T > C (p. K601E) BRAF mutations.

There were five patients (1.3%) who had positive results for BRAF PNA clamping, real-time PCR and immunohistochemistry. For these five patients, direct Sanger sequencing was performed. The results of Sanger sequencing were the same with those of real-time PCR and immunohistochemistry (Figure 2). Considering the PNA clamping method is a very sensitive method to detect a low allele level of mutation, direct sequencing using a clamping PCR product was performed [10]. Regarding the results of sequencing using a clamping PCR product, all cases showed mutation for the BRAF gene other than the V600E genotype (Figure 3). Finally, there were five mutated cases (1.3%) for BRAF in the total cohort. Among them, three cases (0.8%) had a V600E mutation and the other two had V600K and V600V/V601E mutations, respectively. Among the total number of BRAF mutations, V600E genotype was present in three cases, comprising 60% of the BRAF mutant. 

### 3.5. Clinicopathologic Aspects of BRAF Mutation in Lung Cancers

There were three cases (0.8%) of the BRAF V600E mutation among 378 non-small cell carcinomas. It was 1.2% among 255 adenocarcinoma and 246 non-small cell carcinomas without EGFR/ALK aberrations. In addition, it was 2.3% among 129 adenocarcinomas without EGFR/ALK aberrations. There were two cases of BRAF mutation other than V600E, comprising 0.5% of all non-small cell carcinoma, 0.8% of adenocarcinoma and non-small cell carcinoma without EGFR/ALK aberrations and 1.5% of adenocarcinoma without EGFR/ALK aberrations (Table 6).

Among the V600E mutated patients, one patient was a never-smoker and the other two were ever-smokers. There was a micropapillary component in all the V600E-mutated cases (Table 7). All patients harboring a BRAF mutation had no concomitant EGFR or ALK aberrations. Detailed clinicopathologic characteristics of individual patients with a BRAF mutation are listed in Table 7.

## 4. Discussion

In this study, the *BRAF* V600 mutation incidence was found in five patients (1.3%) among all cases of non-small cell carcinoma, and the *BRAF* V600E mutation was present in three patients (0.8%) with adenocarcinoma. It is relatively low when compared to most reports from the Western population [11,12,13,14]. On the other hand, the incidence is similar to that of Japanese patients [15]. In this study, there were 2.3% and 1.5% of the *BRAF* V600E mutation and *BRAF* V600 non-E mutation, respectively, among adenocarcinoma without *EGFR/ALK* alterations. According to one Korean dataset, there were four patients (1.8%) with a *BRAF* mutation among 222 Stage III/IV lung adenocarcinoma patients without *EGFR/ALK* aberrations [16]. The difference from these data probably resulted from the difference in stage distribution of the study cohort. This study included 305 cases (80.7%) of early-stage (Stage I and II) disease, contrary to their advanced stage cohort. This study included 123 cases of non-adenocarcinoma patients, and none of these patients harbored a *BRAF* mutation. However, other data reported the detection of a *BRAF* mutation in non-adenocarcinoma patients, though the incidence was very low [11,13,14,15,17]. Among five *BRAF* V600-mutated lung cancer patients, two patients (40%) were never-smokers and three (60%) were ever-smokers. This is in accordance with the molecular testing guideline for the selection of patients with lung cancer for treatment, suggesting that *BRAF* mutational testing should be performed on all advanced adenocarcinoma patients, irrespective of clinical characteristics [18].

*BRAF* V600 non-E mutation was present in two cases. However, the result of direct sequencing showed the wild type of *BRAF* using amplified DNA extracted from FFPE blocks following sequencing using PNA clamping product detected mutation. Through PNA clamping PCR, the wild-type alleles are inhibited in the amplification process by hybridization with PNA, resulting in mutant enrichment. Though detected mutation was not the V600E genotype in this study, this result suggests that sequencing using the PNA clamping PCR product can help the detection of a mutant of low level in suspected or equivocal cases. In addition, Zengarini et al. presented some treatment effects on *BRAF* V600K mutated melanoma patients [19], and there is also an ongoing phase 2 clinical trial on the application of dabrafenib and trametinib in tumors with the *BRAF* V600E or V600K mutation including non-small cell lung cancer (ClinicalTrials.gov Identifier: NCT04439292). Additionally, *BRAF* V600E real-time PCR showed both 100% of sensitivity and specificity. All these results are in accordance with the principles of molecular and biomarker analysis for *BRAF* by NCCN guideline: “Real-time PCR, Sanger sequencing (ideally paired with tumor enrichment), and NGS are the most commonly deployed methodologies for examining *BRAF* mutation status” (NCCN Guidelines Version 3.2023). 

All patients harboring the *BRAF* V600 mutation had adenocarcinoma and all patients with the V600E genotype had a micropapillary component. The result is similar to those of prior reports [9,20,21]. Theis may suggest that lung adenocarcinoma with micropapillary should be first considered to conduct *BRAF* mutation testing. 

The VE1 mouse monoclonal antibody was utilized in this study. VE1 antibody is a mutation-specific antibody able to differentiate a V600E-mutated protein from wild-type and other *BRAF*-mutated proteins [22]. In this study, immunohistochemistry for VE1 showed both 100% of sensitivity and specificity. Gow et al. validated the usefulness of the Ventana VE1 antibody in lung cancer [20]. They reported that immunohistochemistry for VE1 antibody showed a 96.6% sensitivity to detect the *BRAF* V600E mutation and a 98.6% specificity to discriminate tumors without the *BRAF* V600E mutation. However, one positive case affecting the specificity value had weak positive cytoplasmic staining in 5% of tumor cells and the case had *ROS1* gene fusion. According to their criteria, the case was considered to be negative. Ilie et al. reported that VE1 immunohistochemistry is specific and sensitive to detect the *BRAF* V600E mutation [9]. Similar results were shown by Hofman et al., suggesting that VE1 staining is a rapid, specific and very sensitive method [23]. In addition, Chang et al. reported that VE1 immunohistochemistry showed almost perfect interobserver agreement, suggesting that this could be a screening test for *BRAF* mutation [24]. In present study, *BRAF* V600E mutated cases showed diffuse (100% of proportion) positivity, though the intensity was heterogeneous in two cases. Overall, these results suggest that immunohistochemistry for the Ventana VE1 antibody can be a useful screening tool in lung cancers, especially for small biopsy specimens, which must be handled with care to obtain the maximum information for treatment choice. Moreover, immunohistochemistry has many advantages over molecular diagnostics, namely because it needs much less tissue and the turn-around time is far shorter. 

There are limitations in this study. The detection rate of *BRAF* mutation was only 1.3% of the study cohort, so statistical analyses could not be performed. In addition, these data are from one single institution, which makes it difficult to generalize these findings. However, this study cohort was composed of only the Korean population, and for all experiments, we only used consecutive resected samples of primary lung cancer.

## 5. Conclusions

*BRAF* V600 mutation status in resected primary non-small cell carcinoma was tested. There were five cases (1.3%) of a *BRAF* V600 mutation among 378 non-small cell carcinomas, comprising three cases of a *BRAF* V600E mutation and two cases of a *BRAF* V600 non-E mutation. All cases harboring a *BRAF* V600 mutation were adenocarcinoma without *EGFR* mutation and *ALK* translocation. All three cases of a *BRAF* V600E mutation had micropapillary component. Immunohistochemistry for Ventana VE1 antibody can be a useful screening method to detect a *BRAF* V600E mutation. 

This study preliminarily suggests that the incidence of a *BRAF* V600E mutation might be low in Korean population. In addition, adenocarcinoma showing micropapillary component, especially without *EGFR/ALK* aberration, should be first considered for *BRAF* testing, including immunohistochemistry.

## Figures and Tables

**Table 1:** Clinical characteristics of study population.

Characteristics	Number (%)
Age (years)	66.84 ± 8.76
Sex	
Male	238 (63.0)
Female	140 (37.0)
Smoking status	
Never-smoker	168 (44.4)
Current smoker	98 (25.9)
Ex-smoker	112 (29.6)
Pack-years among ever-smokers (years) *	34.68 ± 19.81
Tumor size (cm)	3.37 ± 1.55
Histologic type	
ADC	255 (67.5)
SqCC	91 (24.1)
SC	15 (4.0)
LCNEC	9 (2.4)
ADSqCC	5 (1.3)
Other	3 (0.8)
Differentiation	
WD	19 (5.0)
MD	269 (71.2)
PD	90 (23.8)
Stage	
Early (I–II)	305 (80.7)
Advanced (III–IV)	73 (19.3)
*EGFR* mutation	
Absent	258 (68.3)
Present	120 (31.7)
*ALK* translocation	
Absent	367 (97.1)
Present	11 (2.9)
Ethnicity Korean	378 (100.0)

* Among the ever-smokers, pack-year data were unavailable in six cases. ADC, Adenocarcinoma; SqCC, Squamous cell carcinoma; SC, sarcomatoid carcinoma; LCNEC, Large-cell neuroendocrine carcinoma; ADSqCC, Adenocasquamous cell carcinoma WD, Well-differentiated; MD, Moderately differentiated; PD, Poorly differentiated; EGFR, epidermal growth factor receptor; ALK, anaplastic lymphoma kinase.

**Table 2:** The results of BRAF mutation according to BRAF mutation assay.

Case Number	*BRAF* V600 PNA Clamping	*BRAF* V600E Real-Time PCR	IHC for VE1	Direct Sanger Sequencing	Direct Sanger Sequencing of the Clamping PCR Product
142	+	+	+	V600E	Not done
270	+	–	–	WT	V600K
324	+	–	–	WT	V600V, K601E
348	+	+	+	V600E	Not done
358	+	+	+	V600E	Not done

*BRAF*, B-type Raf kinase; IHC, immunohistochemistry; PNA, peptide nucleic acid; PCR, polymerase chain reaction; WT, Wild-type; +, positive; −, negative.

**Table 3:** The results of *BRAF* V600 on PNA-clamping.

Case Number	DNA Loading (ng)	Cycle Threshold (C_T_)		ΔC_T_-2	ΔC_T_-1
		Non-PNA	V600	V600	V600
Clamping Control		24.38	36.33	11.95	−1.33
Positive Control		24.03	30	5.97	5
142	10	28.59	31.7	3.11	3.3
270	10	26.91	32.44	5.53	2.56
324	10	25.98	31.82	5.84	3.18
348	10	26.02	30.3	4.28	4.7
358	10	26.21	31.46	5.26	3.54
142	25	27.15	31.13	3.97	3.87
270	25	26.31	32.42	6.1	2.58
324	25	25.15	31.89	6.74	3.11
348	25	25.22	29.6	4.38	5.4
358	25	25.26	31.02	5.76	3.98

*BRAF*, B-type Raf kinase; PNA, peptide nucleic acid, PC, positive control, ΔC_T_ = standard C_T_—sample C_T_.

**Table 4:** The results of *BRAF* V600E on real-time PCR.

Patient Number	Internal Control C_T_	Sample C_T_	Result
142	25.9	31.9	+
270	24.6	NA	−
324	26.6	NA	−
348	24.7	29.6	+
358	24.4	30.6	+

*BRAF*, B-type Raf kinase; C_T_, cycle threshold; NA, not applicable; +, positive; −, negative.

**Table 5:** Immunohistochemistry results of patients who were V600E-positive on either or both *BRAF* PNA-clamping and *BRAF* real-time PCR.

Case Number	Proportion of Cytoplasm Positive Rate	Intensity Pattern	Intensity Scores of Tumor Cells	Result
142	100	Heterogeneous	3+: 40% 2+: 50% 1+: 10% 0: 0%	+
270	0	Homogeneous	3+: 0% 2+: 0% 1+: 0% 0: 100%	−
324	0	Homogeneous	3+: 0% 2+: 0% 1+: 0% 0: 100%	−
348	100	Homogeneous	3+: 100% 2+: 0% 1+: 0% 0: 0%	+
358	100	Heterogeneous	3+:70% 2+:30% 1+: 0% 0: 0%	+

*BRAF*, B-type Raf kinase; +, positive; −, negative.

**Table 6:** Incidence of BRAF mutations.

	*BRAF* Mutation	*BRAF* V600E Mutation	*BRAF* Non-V600E Mutation
	N (%)	N (%)	N (%)
NSCLC (*n* = 378)	5 (1.3)	3 (0.8)	2 (0.5)
Adenocarcinomas (*n* = 255)	5 (2.0)	3 (1.2)	2 (0.8)
NSCLC lacking *EGFR* mutation and *ALK* translocation (*n* = 246)	5 (2.0)	3 (1.2)	2 (0.8)
Adenocarcinomas lacking *EGFR* mutation and *ALK* translocation (*n* = 129)	5 (3.8)	3 (2.3)	2 (1.5)

**Table 7:** Clinicopathological characteristics of individual patients with BRAF mutations.

Case No.	BRAF Mutation	Age (Years)	Sex	Smoking Status	Pack Years	Predominant Histological Subtype	Present of Micropapillary Component	pT Size (cm)	pN Stage	pM Stage	Stage
142	V600E	72	Male	Current	50	Solid	+	6.5	2	0	IIIA
270	V600K	83	Male	Current	15	Acinar	-	3.6	0	0	IB
324	V600V, K601E	67	Female	Never	0	Acinar	-	1.6	0	0	IA
348	V600E	51	Female	Never	0	Acinar	+	2.2	0	0	IA
358	V600E	52	Male	Ex-smoker	7.5	Acinar	+	1.8	0	0	IA

## Data Availability

Not applicable.

## References

[1] Yan N., Guo S., Zhang H., Zhang Z., Shen S., Li X. (2022). BRAF-Mutated Non-Small Cell Lung Cancer: Current Treatment Status and Future Perspective. Front. Oncol..

[2] Raman M., Chen W., Cobb M.H. (2007). Differential regulation and properties of MAPKs. Oncogene.

[3] Forbes S.A., Bindal N., Bamford S., Cole C., Kok C.Y., Beare D., Jia M., Shepherd R., Leung K., Menzies A. (2011). COSMIC: Mining complete cancer genomes in the Catalogue of Somatic Mutations in Cancer. Nucleic Acids Res..

[4] Tabbò F., Pisano C., Mazieres J., Mezquita L., Nadal E., Planchard D., Pradines A., Santamaria D., Swalduz A., Ambrogio C. (2022). How far we have come targeting BRAF-mutant non-small cell lung cancer (NSCLC). Cancer Treat. Rev..

[5] Leonetti A., Facchinetti F., Rossi G., Minari R., Conti A., Friboulet L., Tiseo M., Planchard D. (2018). BRAF in non-small cell lung cancer (NSCLC): Pickaxing another brick in the wall. Cancer Treat. Rev..

[6] Riudavets M., Cascetta P., Planchard D. (2022). Targeting BRAF-mutant non-small cell lung cancer: Current status and future directions. Lung Cancer.

[7] Owsley J., Stein M.K., Porter J., In G.K., Salem M., O’Day S., Elliott A., Poorman K., Gibney G., VanderWalde A. (2021). Prevalence of class I-III BRAF mutations among 114,662 cancer patients in a large genomic database. Exp. Biol. Med. (Maywood).

[8] Mazieres J., Cropet C., Montané L., Barlesi F., Souquet P.J., Quantin X., Dubos-Arvis C., Otto J., Favier L., Avrillon V. (2020). Vemurafenib in non-small-cell lung cancer patients with BRAF. Ann. Oncol..

[9] Ilie M., Long E., Hofman V., Dadone B., Marquette C.H., Mouroux J., Vignaud J.M., Begueret H., Merlio J.P., Capper D. (2013). Diagnostic value of immunohistochemistry for the detection of the BRAFV600E mutation in primary lung adenocarcinoma Caucasian patients. Ann. Oncol..

[10] Kang S.H., Pyo J.Y., Yang S.W., Hong S.W. (2013). Detection of BRAF V600E mutation with thyroid tissue using pyrosequencing: Comparison with PNA-clamping and real-time PCR. Am. J. Clin. Pathol..

[11] Luk P.P., Yu B., Ng C.C., Mercorella B., Selinger C., Lum T., Kao S., O’Toole S.A., Cooper W.A. (2015). BRAF mutations in non-small cell lung cancer. Transl. Lung Cancer Res..

[12] Paik P.K., Arcila M.E., Fara M., Sima C.S., Miller V.A., Kris M.G., Ladanyi M., Riely G.J. (2011). Clinical characteristics of patients with lung adenocarcinomas harboring BRAF mutations. J. Clin. Oncol..

[13] Marchetti A., Felicioni L., Malatesta S., Grazia Sciarrotta M., Guetti L., Chella A., Viola P., Pullara C., Mucilli F., Buttitta F. (2011). Clinical features and outcome of patients with non-small-cell lung cancer harboring BRAF mutations. J. Clin. Oncol..

[14] Tissot C., Couraud S., Tanguy R., Bringuier P.P., Girard N., Souquet P.J. (2016). Clinical characteristics and outcome of patients with lung cancer harboring BRAF mutations. Lung Cancer.

[15] Kinno T., Tsuta K., Shiraishi K., Mizukami T., Suzuki M., Yoshida A., Suzuki K., Asamura H., Furuta K., Kohno T. (2014). Clinicopathological features of nonsmall cell lung carcinomas with BRAF mutations. Ann. Oncol..

[16] Kim H.C., Kang Y.R., Ji W., Kim Y.J., Yoon S., Lee J.C., Choi C.M. (2019). Frequency and clinical features of BRAF mutations among patients with stage III/IV lung adenocarcinoma without EGFR/ALK aberrations. Onco Targets Ther..

[17] Brustugun O.T., Khattak A.M., Trømborg A.K., Beigi M., Beiske K., Lund-Iversen M., Helland Å. (2014). BRAF-mutations in non-small cell lung cancer. Lung Cancer.

[18] Kalemkerian G.P., Narula N., Kennedy E.B., Biermann W.A., Donington J., Leighl N.B., Lew M., Pantelas J., Ramalingam S.S., Reck M. (2018). Molecular Testing Guideline for the Selection of Patients With Lung Cancer for Treatment With Targeted Tyrosine Kinase Inhibitors: American Society of Clinical Oncology Endorsement of the College of American Pathologists/International Association for the Study of Lung Cancer/Association for Molecular Pathology Clinical Practice Guideline Update. J. Clin. Oncol..

[19] Zengarini C., Mussi M., Veronesi G., Alessandrini A., Lambertini M., Dika E. (2022). BRAF V600K vs. BRAF V600E: A comparison of clinical and dermoscopic characteristics and response to immunotherapies and targeted therapies. Clin. Exp. Dermatol..

[20] Gow C.H., Hsieh M.S., Lin Y.T., Liu Y.N., Shih J.Y. (2019). Validation of Immunohistochemistry for the Detection of. Cancers.

[21] Hwang I., Choi Y.L., Lee H., Hwang S., Lee B., Yang H., Chelakkot C., Han J. (2022). Selection Strategies and Practical Application of BRAF V600E-Mutated Non-Small Cell Lung Carcinoma. Cancer Res. Treat..

[22] Capper D., Preusser M., Habel A., Sahm F., Ackermann U., Schindler G., Pusch S., Mechtersheimer G., Zentgraf H., von Deimling A. (2011). Assessment of BRAF V600E mutation status by immunohistochemistry with a mutation-specific monoclonal antibody. Acta Neuropathol..

[23] Hofman V., Benzaquen J., Heeke S., Lassalle S., Poudenx M., Long E., Lantéri E., Bordone O., Lespinet V., Tanga V. (2020). Real-world assessment of the BRAF status in non-squamous cell lung carcinoma using VE1 immunohistochemistry: A single laboratory experience (LPCE, Nice, France). Lung Cancer.

[24] Chang S., Choi Y.L., Shim H.S., Lee G.K., Ha S.Y., Group K.C.P.S. (2022). Usefulness of BRAF VE1 immunohistochemistry in non-small cell lung cancers: A multi-institutional study by 15 pathologists in Korea. J. Pathol. Transl. Med..

